# The dimerization interface in VraR is essential for induction of the cell wall stress response in *Staphylococcus aureus*: a potential druggable target

**DOI:** 10.1186/s12866-019-1529-0

**Published:** 2019-07-05

**Authors:** Ghazal Tajbakhsh, Dasantila Golemi-Kotra

**Affiliations:** Department of Biology, York University, Toronto, ON M3J1P3 Canada

**Keywords:** Two-component systems, VraSR, Cell wall stress stimulon, *Staphylococcus aureus*

## Abstract

**Background:**

*Staphylococcus aureus* remains a medical challenge in the treatment of bacterial infections. It has acquired resistance to commonly used antibiotics, and to those considered to be the last weapons in treating staphylococcal infections, such as vancomycin. Studies have revealed that *S. aureus* is capable of mounting a rapid response to antibiotics that target cell wall peptidoglycan biosynthesis, such as β-lactams and vancomycin. The two-component system VraSR has been linked to the coordination of this response. VraS is a histidine kinase that undergoes autophosphorylation in the presence of signals elicited upon cell wall damage and it then transfers its phosphoryl group to VraR. VraR is a response regulator protein that functions as a transcription factor. Phosphorylation of VraR leads to its dimerization, which is required for optimum binding to its target promoters. Two-component systems have been targeted for the development of antibacterial agents. Deletion of the *vraS* or *vraR* gene has been shown to re-sensitize *S. aureus* to β-lactams and vancomycin.

**Results:**

In this study, we explored perturbation of the VraR phosphorylation-induced activation as a means to inhibit the VraSR-mediated signal transduction pathway. We show that dimerization of VraR is essential for the phosphorylation-induced activation of VraR. A single point mutation in the dimerization interface of VraR, in which Met13 was replaced by Ala, led to the inability of VraR to dimerize and to bind optimally to the target promoter. The consequences of these in vitro molecular deficiencies are equally dramatic in vivo. Complementation of a *vraR* deletion *S. aureus* strain with the *vraRM13Ala* mutant gene failed to induce the cell wall stress response.

**Conclusions:**

This study highlights the potential of targeting the phosphorylation-induced dimerization of VraR to disrupt the *S. aureus* cell wall stress response and in turn to re-sensitize *S. aureus* to β-lactams and vancomycin.

**Electronic supplementary material:**

The online version of this article (10.1186/s12866-019-1529-0) contains supplementary material, which is available to authorized users.

## Background

**Fig. 1:**
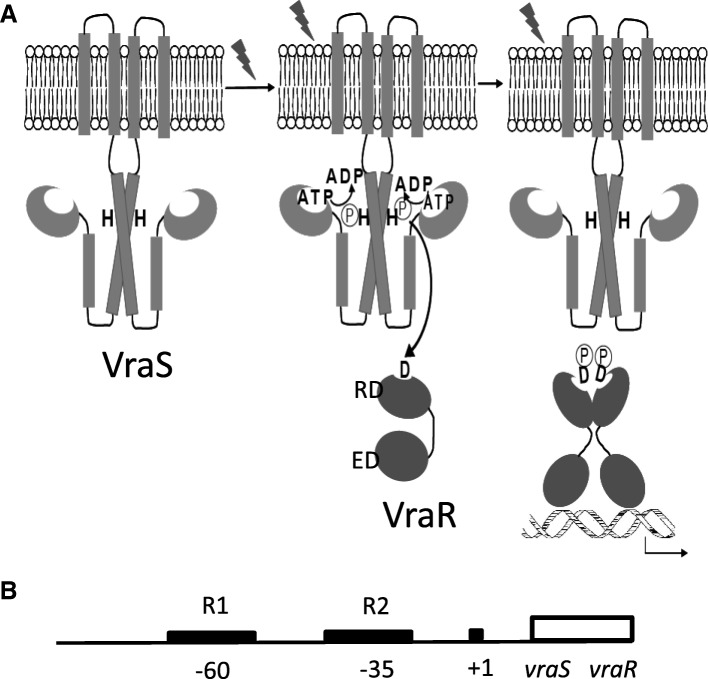
Schematic views of the signal transduction pathway mediated by VraSR (**a**) and of the VraR-bindinding sites on the vraSR promoter (**b**)

Two-component signaling systems (TCSs) are prevalent in bacteria [1, 2]. They enable coupling of a diverse array of adaptive responses to environmental stimuli including antibiotic stress [1, 3]. Their absence in high eukaryotic systems makes them a prime target for development of novel antimicrobial agents [4]. Signaling via TCSs is based on a conserved phosphor transfer process between a histidine kinase (HK) and a response regulator protein (RR) (Fig. 1). The output response is controlled by the RR, which plays the role of a phosphorylation-activated switch [5]. About 60% of all identified RRs act as transcription factors [5].

The vancomycin-resistance-associated response regulator protein, VraR, and its cognate histidine kinase, VraS, constitute a two-component system in *Staphylococcus aureus* (Fig. 1a) that has been shown to mediate resistance to β-lactams and vancomycin [6]. VraSR has gained attention as a potential target in encountering the antibiotic resistance in multiple antibiotic resistant *S. aureus* strains such as MRSA. Deletions within the *vraSR* operon re-sensitize MRSA *strains* to β-lactams and vancomycin [7–11]. The VraSR-mediated signal transduction pathway is activated by the exposure of *S. aureus* to antibiotics and gene deletions that inhibit cell wall synthesis [6, 12, 13]. Cell wall damage in *S. aureus* leads to the VraR-dependent regulation of more than 40 genes [6]. However, the precise nature of the signal(s) that turns on the VraSR-mediated signaling pathway is not known. Both VraS and VraR are multidomain proteins (Fig. 1a). VraS consists of an N-terminal transmembrane domain, which, in a number of histidine kinases, is involved in sensing the extracellular stimuli, and a C-terminal core domain that harbors the dimerization interface, the conserved histidine residue, and the ATP-binding domain. VraR consist of a conserved N-terminal domain that harbors the phosphorylation site, referred to as the regulator domain (RD), and a variable C-terminal domain that interacts with DNA, referred to as the effector domain (ED) (Fig. 1a). Upon cellular stress, VraS is activated through an autophosphorylation event (*t*_1/2_ ~ 10 min) [12, 14]. Subsequently, the histidine kinase transfers the phosphoryl group, almost instantaneously (*t*_1/2_ ~ 10 s), onto VraR [14]. Upon its phosphorylation, VraR dimerizes at the N-terminal domain. Phosphorylation-induced dimerization is shown to expand and enhance the VraR binding to its own promoter (Fig. 1b) [14–16], and, consequently, to increase the expression of the *vraSR* operon [17]. Higher *vraR* expression leads to modulation of as many as 40 genes, which ultimately constitute the *S. aureus* response to cell wall damage. The VraSR-mediated signal transduction is reset in the absence of stress through the phosphatase activity of VraS toward VraR [14].

**Fig. 2:**
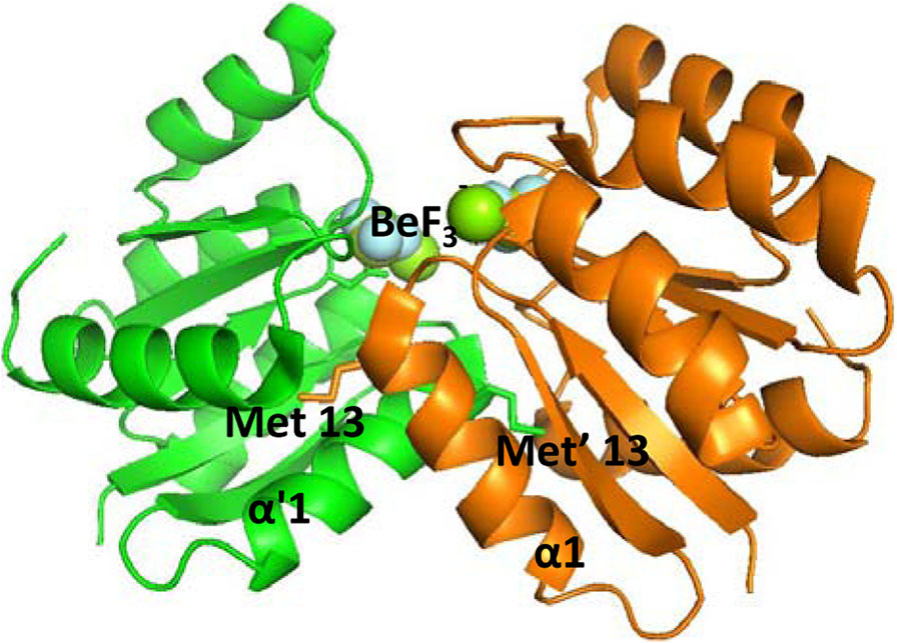
The dimerization interface as resolved by X-Ray crystallography (PDB ID: 4IF4)

The events that lead to the phosphorylation-induced dimerization of VraR have been elucidated by hydrogen-deuterium mass spectrometry and X-ray crystallography [16, 18]. These studies showed occurrence of structural rearrangement in the VraR regulator domain in the phosphorylated dimeric species. In particular, the crystal structure showed that these structural rearrangements ultimately are associated with the formation of a hydrophobic pocket that wraps snugly the side chain of a methionine residue, Met13, which protrudes from an α-helix region (α1 helix) of the opposing protomer in the dimer complex (Fig. 2) [16]. This discovery provided a strategy for the targeting of two-component signal transduction pathways, that of inhibition of the phosphorylation-induced dimerization of RR.

VraR belongs to the NarL/FixJ family of RR, which use the helix-turn-helix motif to bind to DNA. The dimerization interface found in VraR is seen in a subset of this family of proteins, for example in *Escherichia coli* UhpA [19], a response regulator that regulates sugar uptake, *Mycobacterium tuberculosis* NarL [20], *S. aureus* LuxR (PDB ID 3B2N), a response regulator implicated in regulation of cell-density, *Bacillus subtillis* DesR [21], and *Enterococcus faecium* LiaR, a response regulator involved in daptomycin induced cell wall stress response [22]. The response regulator proteins that belong to the OmpR/PhoB family of proteins, which use a winged helix-turn-helix to target DNA, implicate the α4-β5-α5 interface for dimerization [23].

In this study, we investigated the role of Met13 in the phosphorylation-induced dimerization and binding of VraR to its own promoter. Our in vitro and in vivo studies revealed the profound effect that this single amino acid residue has on the cell-wall antibiotic stress response in *S. aureus*. In addition, they validated the significance of inhibition of the phosphorylation-induced activation of VraR in re-sensitizing *S. aureus* to β-lactams and vancomycin.

## Results

### Characterization of the VraRM13A variant

The VraRM13A variant was purified to homogeneity as described before [14]. To assess the impact of substitution of Met13 by Ala on the overall secondary structures and the structural integrity of VraR, we recorded the CD spectrum of the VraRM13A variant and its thermal melting, respectively, and compared these data against the wild-type protein CD spectrum and the thermal melting. The CD spectra of VraR and VraRM13A were similar, indicating that the substitution of Met by Ala did not have a deleterious effect on the secondary structural features of the protein (see Additional file 1: Figure S1A). This outcome was to be expected as Met-13 residue sits on an α-helix (α1) and its substitute, alanine, is a strong α-helix former [24]. The thermal melting studies also showed that the stability of the variant protein was similar to that of the wild-type protein, inferring that the overall integrity of the protein was not affected (see Additional file 1: Figure S1B).

### Phosphorylation of VraRM13A

**Fig. 3:**
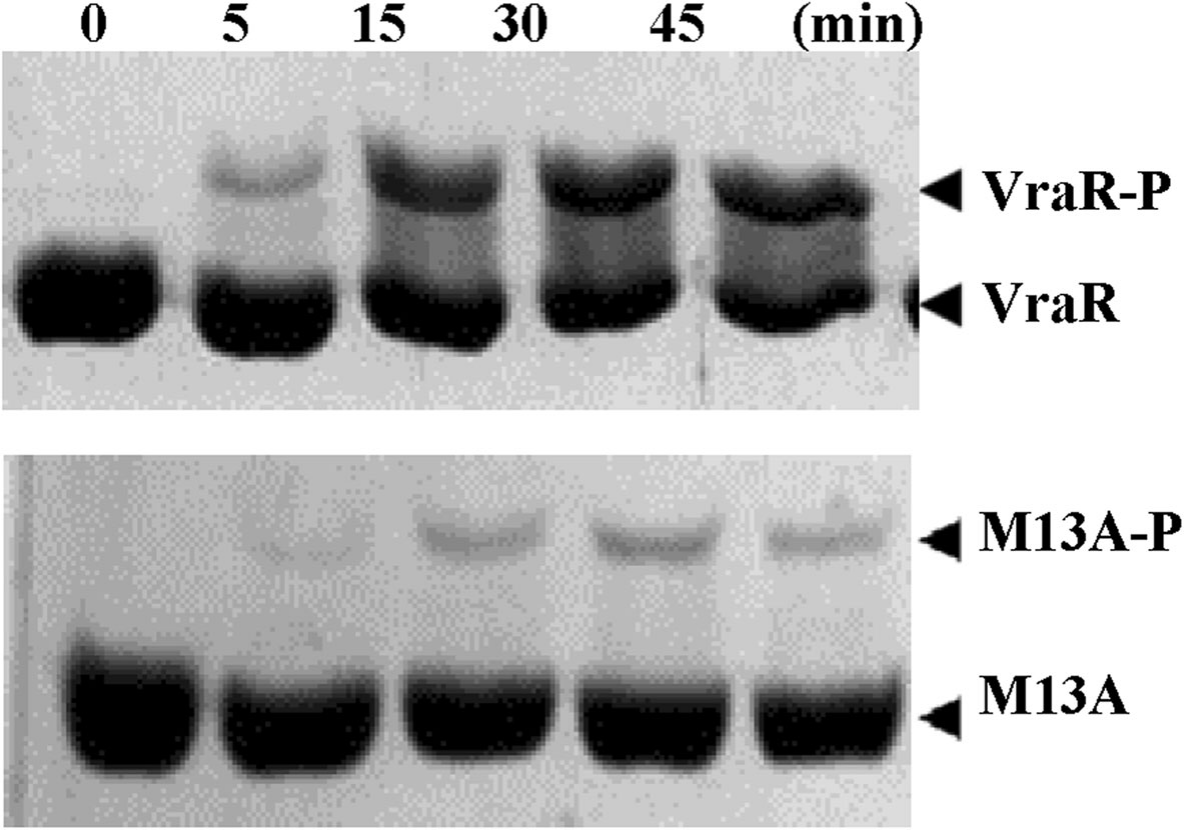
Time-dependence phosphorylation of VraR and VraRM13A (M13A) by acetyl phosphate. Coomassie blue staining of the Phostag 12.5% SDS-PAGE. The experimental conditions were as follows: VraR at 20 μM concentration was incubated with 50 mM acetyl phosphate in PB buffer at 37 °C at different time intervals

Phosphorylation of VraR and VraRM13A by a small molecule donor such as acetyl phosphate was assessed by Phos-tag™ SDS-PAGE: the un-phosphorylated and phosphorylated protein species were separated by the SDS-PAGE as a result of the phosphorylated species mobility being slowed down by the Phostag reagent in the SDS-PAGE (Fig. 3). The protein bands, stained with coomassie blue, were quantified by ImageJ (NIH). These experiments showed that after 45 min of incubation the VraRM13A variant underwent about 10% phosphorylation compared to 73% of phosphorylation measured for VraR.

### Oligomerization state of VraRM13A

**Fig. 4:**
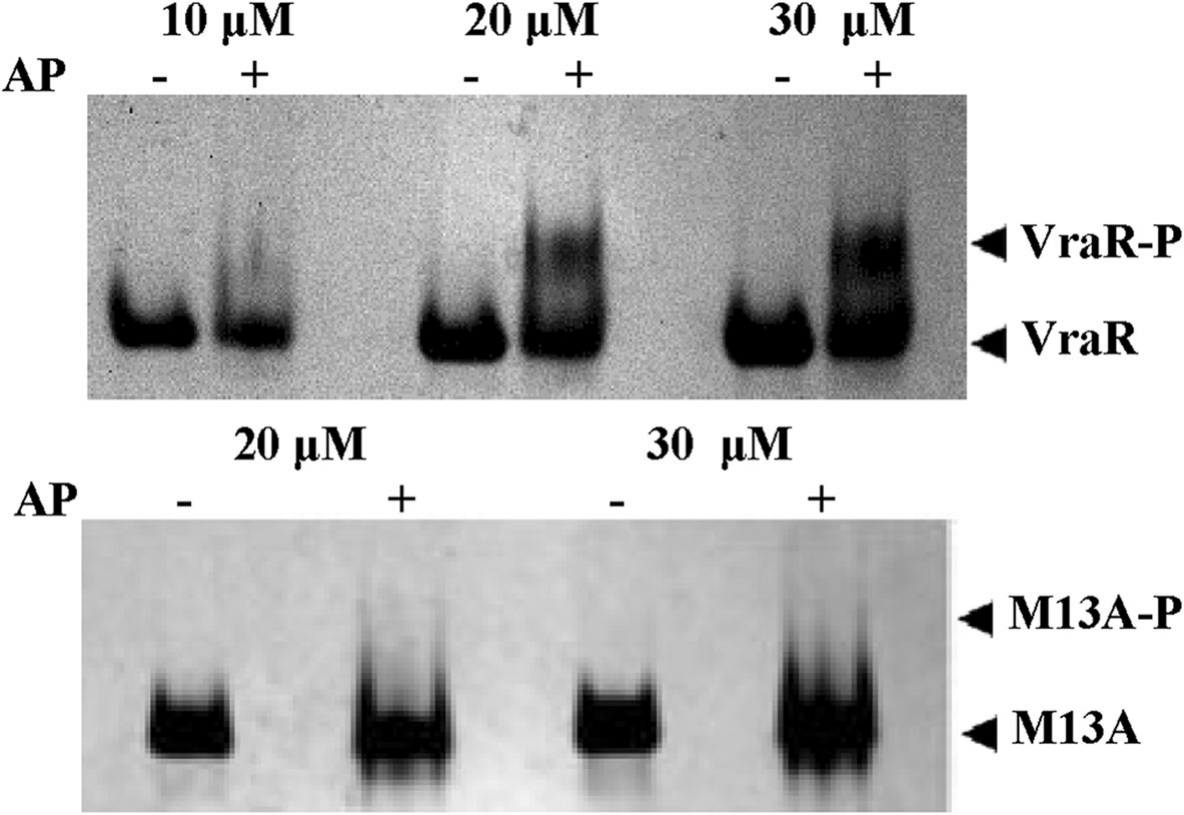
Coomassie blue staining of the 10% Native-PAGE of VraR and VraRM13A (M13A) phosphorylated by acetyl phosphate (AP). Briefly, VraR at the concentrations denoted in the figure was phosphorylated by 50 mM acetyl phosphate in the PB buffer for 1 h, at 37 °C

Earlier studies on VraR have shown that phosphorylation drives the protein, otherwise a monomer, toward dimerization [14]. The Met-13 residue was identified as one of the residues that participated intimately in dimerization (Fig. 2) [16]. Hence, reduction of the methionine side chain to that of a methyl group should have an impact on the dimerization. The native-PAGE experiments were performed to investigate the protein dimerization ability after attempting the phosphorylation. The VraRM13A variant, subjected to the phosphorylation, did not form a dimer at 10 μM, 20 μM or 30 μM (or 40 μM, data not shown), unlike VraR (Fig. 4).

### Analysis of the DNA-binding activity of VraR

**Fig. 5:**
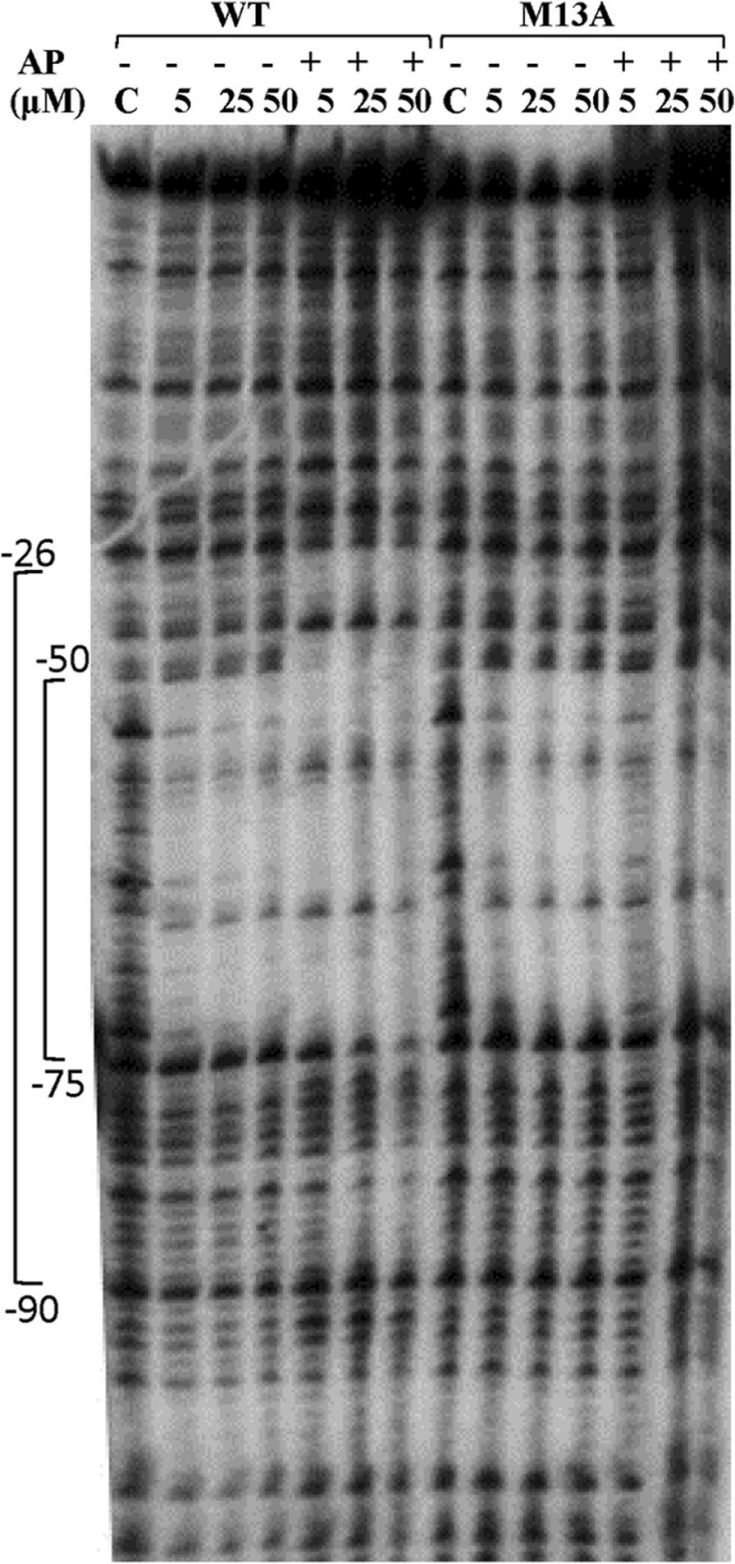
DNase I footprinting experiment. The top strand of P_*vraSR*_ was labelled with ^32^P at the 5′-end, and incubated with VraR, VraR-P, VraMet13Ala (M13A), and VraRM13A subjected to phosphorylation with acetyl phosphate. The sample were each protein was absent from the reaction mixture is denoted by C

To evaluate the DNA-binding activity of VraRM13A, we performed DNase I footprinting experiments. Previous studies showed that unphosphorylated VraR binds to the region − 50 to − 75 (namely R1) of the *vrasR* promoter (P_*VraSR*_) while phosphorylated VraR (VraR-P) binds to an additional site of the promoter, the region − 46 to − 26 (namely R2), (Fig. 1b) [15]. The DNase I footprinting experiments carried out with VraRM13A showed that unphosphorylated and acetyl phosphate-treated VraRM13A bound to P_*VraSR*_ in the same way as unphosphorylated VraR (Fig. 5); only the R1 was occupied by these proteins, not the R2 site. These results are in agreement with the outcome of the phosphorylation and oligomerization experiments, which showed that VraRM13A subjected to phosphorylation with acetyl phosphate failed to undergo quantitative phosphorylation and any measurable dimerization. We analyzed the band intensities of three of the DNA bands observed in the VraR-protected region of P_*VraSR*_ by ImageJ, and compared the DNA binding affinity of VraRM13A to that of VraR (see Additional file 2: Figure S2). The binding isotherms show that binding of VraRM13A to the R1 site of P_*VraSR*_ was weaker than binding of VraR. We had shown earlier that the VraR DNA-binding sequence consists of two in-tandem sequences which in R1 are very close to each other, separated by one nucleotide, and as such they are likely to promote dimerization of VraR in this site [17]. The weaker DNA-binding observed for VraRM13A, in comparison to VraR, could be due to the inability of VraRM13A to engage into a dimer at the R1 site.

### Activation of the *vraR* regulon in the presence of *vraRM13A*

To assess in vivo the effect of the Met13 to Ala substitution on the activity of VraR, we generated the P_*vraSR*_::*vraR*M13A construct and cloned it into the shuttle vector pMK4 [25]. The vector pMK4::P_*vraSR*_*vraR*M13A complemented the *RN4220*Δ*vraR* strain well as assessed by measuring the expression levels of *vraR* in the absence and presence of oxacillin using RT-qPCR (data not shown).

In the current study, we selected a few genes from the *vraR* regulon to assess the effect of the Met to Ala substitution into the transcriptional activity of VraR. These genes were *fmtA*, *sgtB* and *pbpB* [6]. Expressions of these genes in the absence of oxacillin were compared to their respective gene expressions upon exposure of RN4220Δ*vraR*(pMK4::P_*vraSR*_*vraR*M13A) to sub-inhibitory concentration of oxacillin, for 30 min (data not shown). In all the cases, the complementation of RN4220Δ*vraR* with *vraR*M13A variant failed to increase gene expression of *vraR*, *fmtA*, *sgtB* and *pbpB* in response to oxacillin. The lack of upregulation of the above genes in the presence of oxacillin is very likely due to the failure of *vraRM13A* to be induced by oxacillin in the RN4220Δ*vraR*(pMK4::P_*vraSR*_*vraR*M13A) strain.

## Discussion

Substitution of a single residue in VraR, Met13 to Ala, led to the inability of VraR to undergo quantitative phosphorylation by small molecule donors such as acetyl phosphate and to dimerize in vitro. In vivo, complementation of the RN4220*ΔvraR* strain with *vraRM13A* did not lead to a response to oxacillin-induced stress. We had earlier suggested that the dimerization domain in VraR is located at the regulatory domain [14]. The X-ray crystal structure of the VraR dimer showed that the dimerization interface in the active VraR species (BeF^3−^-VraR bound species) is located at the regulator domain of VraR [16]. Peculiarly, at the center of this dimerization interface was the side chain of Met13, sitting on the α1 helix and snugly fitted into a hydrophobic pocket formed on the opposing molecule of VraR in the dimer [16]. In this study, we show that substitution of Met13 with Ala does not have measurable effects on the overall VraR structural integrity as assessed by CD. Thus, any impact on the activity of VraMet13Ala was attributed to the lack of the methionine-13 side chain.

**Fig. 6:**
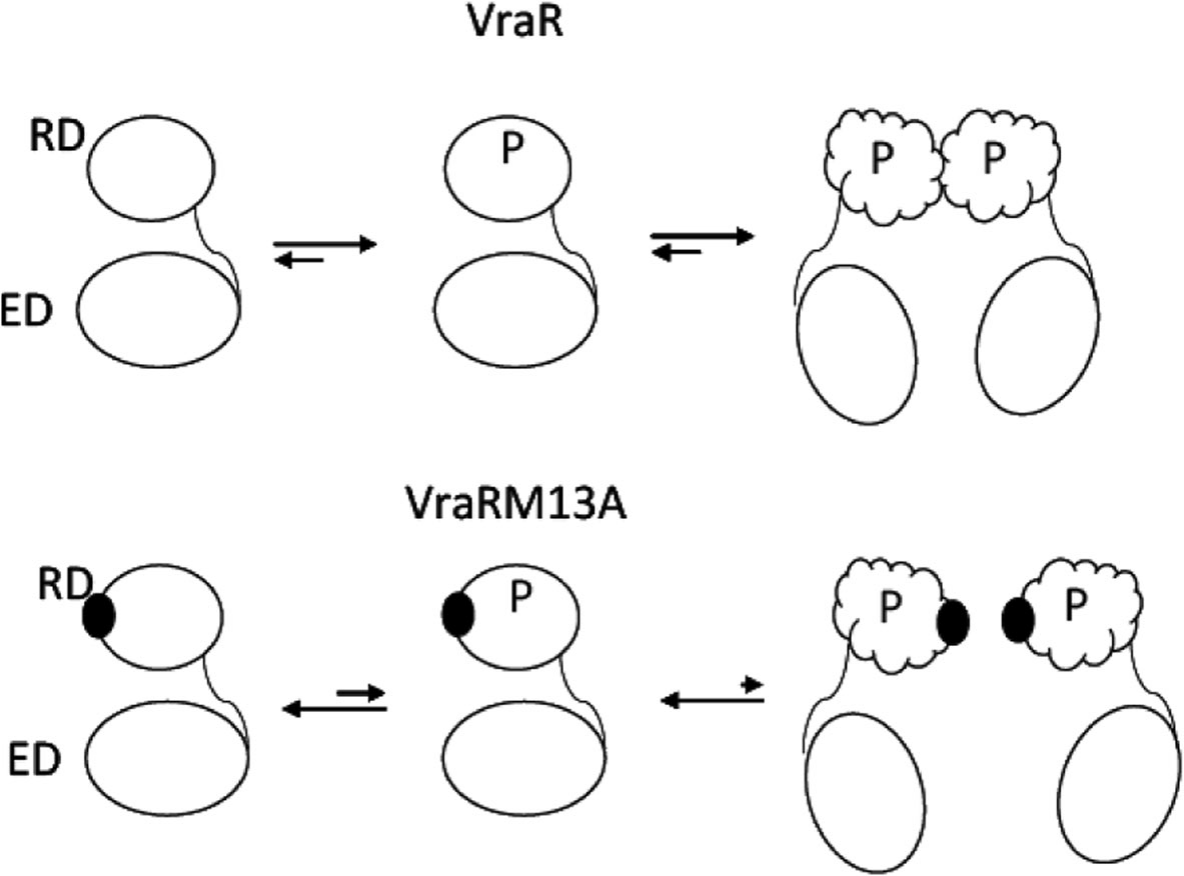
The schematic view of the structural rearrangements that take place upon VraR-P dimerization that lead to the activation of the protein

Autophosphorylation of VraRM13A by a small molecule phosphodonor such as acetyl phosphate was about 7-fold less compared to the wild-type protein. We attributed this outcome to the inability of VraRM13A to dimerize in the absence of the methionine sidechain. Dimerization is considered to be a process that drives forward phosphorylation of RR [26]. The aspartyl phosphate species carry a high negative free energy and dimerization can channel that energy in the form of conformational changes in the protein. These conformational changes, which mark activation of RR, facilitate binding of RR to itself (oligomerization) and/or the target DNA sequence or other proteins [26]. So if dimerization is impeded, the phosphorylation of RR will also be affected (Fig. 6). This explains the slower phosphorylation rate of VraM13A and highlights the significance of the Met-13 residue in dimerization of VraR.

Phosphorylation of VraR is required for upregulation of its own operon, the *vraSR* operon [15, 17]. We had shown earlier that phosphorylation-induced dimerization of VraR is required to bind to a lesser conserved DNA-binding site on the *vraSR* promoter, namely the R2 site (Fig. 1b); while a conserved DNA-binding site, namely the R1 site, in the same promoter, can recruit the unphosphorylated VraR protein (Fig. 1b). In the case of VraRM13A, the DNase I footprinting experiments showed that subjection of this variant protein to phosphorylation did not lead to binding to the R2 site even at protein concentrations as high as 50 μM. However, the conserved DNA-binding site, the R1 site, was occupied by VraRM13A. This result confirms our earlier work that dimerization of VraR is required for recognition of a DNA-binding site that is less conserved [17]. The significance of dimerization in gene activation by VraR was corroborated by the RT-qPCR experiments. Indeed, the wild-type *vraR* gene was able to complement the RN4220Δ*vraR* strain, and mediate the *vraR* regulon in the presence of oxacillin, as indicated by the increased in the expression levels of the *fmtA*, *sgtB* and *pbpB* genes. However, complementation of the RN4220Δ*vraR* strain with *vraRM13A* failed to mediate the *vraR* regulon in the presence of oxacillin, as indicated by the lack thereof of upregulation of the above genes. This observation is very likely due to the lack of upregulation of the *vraSR* operon in the presence of oxacillin. VraR is known to regulate its own operon, hence if VraRM13A is not able to bind to its own promoter optimally it will not lead to higher expression levels of *vraR* in the presence of stress, and subsequently VraR cannot regulate its target genes.

## Conclusions

The impacts of substitution of Met13 with Ala on the phosphorylation rate, dimerization and gene activation by VraR demonstrates the significance of this residue in driving the phosphorylation-induced activation of VraR, and hence cell wall stress response in *S. aureus*. As such, targeting the dimerization interface in VraR, and possibly in other response regulators of the NarL/FixJ family, through small molecules is a strategy that can efficiently disrupt the signal transduction mechanism of bacterial response to their respective stimuli. In addition, due to their conservative nature, inhibition of RR offers a global strategy in targeting a wide range of pathogens. Furthermore, RRs offer a unique and a direct site of inhibition in TCSs; in the past, inhibition of HK did not lead to inactivation of RR due to the ability of RR to cross-talk with other phosphate-donors in the cell and by-pass the histidine kinase [4].

## Methods

Chemicals, growth media, and antibiotics were purchased from Thermo-Fisher (Whitby, Canada) or Sigma (Oakville, Canada), unless otherwise stated. *Escherichia coli* strains, such as NovaBlue and BL21(DE3), and other expression plasmids were purchased from EMD4 Biosciences (New Jersey, USA). All the primers were purchased from Sigma (Oakville, Canada). Chromatography columns and media were bought from GE Healthcare (Mississauga, ON, CA). Radioactive [γ-^32^P]-ATP was purchased from Perkin Elmer (Life and Analytical Sciences, Waltham, MA, USA).

### Generation of the *vraR*M13A variant, production and purification of the VraRM13A protein

The *vraR*M13A variant was generated through site directed mutagenesis using the QuikChange Site-Directed Mutagenesis kit (Agilent). The nucleic acid sequences of the mutagenic primers used in these experiments were as follows: Dir: 5′-GTGGATGATCATGAA*GCG*GTACGTAT AG, Rev.: 5′-*CTATACGTACCGC*TTCATGATCATCCAC. The pET26b::*vraR* vector [14] was amplified using the above mutagenic primer pair as suggested by the vendor. The resulting amplicon was subjected to digestion by Dpn*I* and then used to transform *E. coli* NovaBlue. Mutation was confirmed by DNA sequencing at the Centre for Applied genomics (TCAG) facilities (Sick Kid’s Hospital). The mutated pET26b::*vraR*M13A vector was introduced into the expression host *E. coli* BL21(DE3). The induction, isolation and purification of VraRM13A variant were carried out similarly to that of the wild-type protein, and as described by [14].

### Circular dichroism (CD) spectroscopy and thermal melting of VraRM13A

The CD spectrum of the target protein was carried out by preparing a protein solution of 10 μM in 20 mM Tris, pH 7.0 buffer, and supplemented with 5 mM MgCl2. The CD spectrum was recorded from 200 to 260 nm in a Jasco J-810 instrument (0.1 cm path length cuvette). To investigate the effect of the mutation on the overall structure of the protein, the thermal melting of the protein was carried out by monitoring the CD signal at 222 nm from 20 °C to 100 °C in a Jasco J-810 instrument.

### Phosphorylation of VraR by lithium potassium acetyl phosphate

Phosphorylation of VraR, or VraM13A variant (10 μM or 20 μM), was carried out with 50 mM acetyl phosphate in the phosphorylation buffer (1X PB: 50 mM Tris, pH 7.4 buffer, 50 mM KCl, 20 mM MgCl2). The reaction mixtures were incubated for up to 1 h at 37 °C. The extent of protein phosphorylation was evaluated by Phos-tag™ 12.5% SDS polyacrylamide gel electrophoresis (PAGE) [27].

### Examination of the oligomerization state of VraR and VraM13A by native-PAGE

Dimerization of VraR and its variant upon phosphorylation was examined by native-PAGE. Briefly, the protein at concentrations 10 μM, 20 μM, and 30 μM was phosphorylated as described above and samples were resolved into a 10% native-PAGE at 4 °C.

### DNase I footprinting assay

To perform this assay the region − 121 to + 26 of the *vraSR* promoter (P_***vraSR***_) was amplified by PCR using a ^32^P -5′-end direct primer Dir: 5′-ACGAAGCTTGGTCCGATTTTAACGAC AAAAATTG-3′ and a reverse primer Rev: 5′-TGAAATGACGCATTGATTGTGTTC-3′ [15]. The amplicon (2 ng) was incubated with VraR, phosphorylated VraR (VraR-P), VraRM13A and VraRM13A subjected to phosphorylation (VraRM13A/P) at different concentrations (0, 2, 5, 25, and 50 μM) in the binding buffer (10 mM Tris, pH 7.5 buffer, 50 mM KCL, 1 mM DTT) supplemented with 10 mM MgCl2, 50% glycerol and 20 ng/μl Herring sperm DNA. Each binding reaction was subjected to DNase I for 2 min to digest the DNA and then the DNase I stop solution (1% SDS, 0.2 M NaCl, 20 mM EDTA, pH 8.0) to stop the reaction. Samples were resolved by an 8% polyacrylamide gel containing urea, and the DNA bands were visualized using a Typhoon Trio^+^ Variable Mode Imager (GE HealthCare).

### Construction of the complementing vector, pMK4::P_*vraSR*_*vraR*

In these experiments, the *vraSR* promoter region from − 121 to + 150 (P_*vraSR*_) was fused to the *vraR* gene (P*vraSR::vraR*) and the entire sequence was cloned into the pMK4 shuttle vector in order to complement the *S. aureus* Δ*vraR* strain and assess the effect of the M13A substitution into the VraR activity in vivo*,* by RT-qPCR. Briefly, *P*_*vraSR*_ was amplified using the primer pair Dir: 5’AGGAATTCGGTCCGATTTTAACGACAAAAATTG and Rev.: 5’CGGGATCCAC GTTCAACATAGTTCATAAC (the underlined regions represent the sequences of the restriction enzyme sites, EcoR*I* and BamH*I* respectively) and the *vraR* gene was amplified using the primer pair Dir: 5’CGGGATCCATGACGATTAAAGTATTGTTTG and Rev.: *5’G*CGTCGA*C*CTAT TGAATTAAATTATGTTG (the underlined regions represent the sequences of the restriction enzyme sites, BamH*I* and Sal*I* respectively). The P_*vraSR*_::*vraR* construct was ligated into the pMK4 vector at the EcoR*I* and *S*al*I* restriction sites. The sequence of the construct was confirmed by DNA sequencing. The pMK4::P_*vraSR*_*vraR* plasmid was introduced to *S. aureus* RN4220Δ*vraR* competent cells by electroporation (2 kV, 2.5 ms) using Micropulser (Bio-Rad) and were grown on TSB agar supplemented by 10 μg/ml chloramphenicol. Introduction of the mutation on the *vraR* gene at the pMK4::P_*vraSR*_*vraR* plasmid was carried out by site-directed mutagenesis as outlined above.

### RT-qPCR to investigate expression of the *vraR* regulon genes in the RN4220Δ*vraR* strain complemented with *vraR*M13A

An overnight seed culture of one of the RN4220 strains was prepared in TSB media supplemented by 10 μg/ml chloramphenicol. An aliquot of 160 μl of overnight seed culture were used to inoculate 16 ml TSB medium supplemented with 10 μg/ml chloramphenicol. The subsequent culture was incubated at 37 °C (200 rpm) up to an optical density at 600 nm (OD_600_) of 0.4. The culture was split into two 5 ml aliquots and one of them was treated with 10 μg/ml oxacillin which was incubated along with the control sample, namely the non-oxacillin treated sample, at 37 °C for 30 min. An aliquot of 1 ml growth culture was used to isolate RNA using the RNeasy Mini kit (Qiagen) as per vendor’s instructions.

High Capacity RNA-to-cDNA kit (Life Technologies) was used to synthesize cDNA from 500 ng of DNase I treated RNA. The 16 s RNA gene was used as an internal control using designed primers: Dir-5’GCTAAGTGTTAGGGGGTTTCC and Rev-5’TTCAACCTTGCGGTCGTACT. The 20 μl reaction mixtures consisted of 25 ng of cDNA, 0.25 μM of each primer (accordingly designed to target specific gene, Table 1), and 10 μl of SYBRE SELECT Master Mix (Life Technologies). The Rotor-gene Q qRT-PCR cycler (Qiagen) was used to amplify the cDNA. The PCR conditions were as follows: First hold: 2 min at 50 °C, second hold:10 min for 95 °C, 40 cycles of 95 °C for 15 s, 60 °C for 30 s, and 72 °C for 30 min; and 72 °C for 10 min as a final extension step. The sequences of the primers that were used to probe the expression of genes such as *vraR*, *fmtA, sgtB* and *pbpB* are provided in Table 1.

**Table 1:** The DNA sequence of the primers used in the RT-qPCR studies

Primer name	Primer Sequence 5′ > 3′
*vraR*Dir	TTTGAACCGGAAGTTTTAGTG
*vraR*Rev	TCCATTTCTCGTTCTGTAAGC
*sgtB*Dir	CCTTTCAAATCGAATCCATGA
*sgtB*Rev	TCAGCTGATAACATGCCAGAG
*fmtA*Dir	TGGTACGAAAAAGTATCCAGATG
*fmtA*Rev	CCAAAGAATCCCCCGTTAAG
*pbp2*Dir	GAACATGGCGCACTTGATTA
*pbp2*Rev	GAGGCACCTTCAGAACCAAA

## Additional files

Additional file 1: CD spectra. CD spectra of VraR and VraRM13A, and their respective thermal melting graphs. (PDF 57 kb)

Additional file 2: DNase I footprinting. Binding isotherms extracted from the DNase I footprinting experiments. (PDF 45 kb)

## Data Availability

The datasets supporting the conclusions of this article are included within the article and/or additional supporting files, with the exception of the RT-qPCR data. The RT-qPCR data are available from the corresponding author on reasonable request.

## Abbreviations

ATP: Adenosine triphosphate
CD: Circular dichroism
EMSA: Electromobility shift assay
PAGE: Polyacrylamide gel electrophoresis
PCR: Polymerase chain reaction
RT: Reverse transcription
SDS: Sodium dodecyl sulphate
VraR: Vancomycin-resistance-associated response regulator protein
