# Supplementary material for: The dimerization interface in VraR is essential for induction of the cell wall stress response in Staphylococcus aureus: a potential druggable target
Source: BMC Microbiol. 2019 Jul 5;19:153. doi: 10.1186/s12866-019-1529-0 (PMC6612188; doi:10.1186/s12866-019-1529-0)
Supplement: Supplementary file 1 — CD spectra. CD spectra of VraR and VraRM13A, and their respective thermal melting graphs. (PDF 57 kb) [file 12866_2019_1529_MOESM1_ESM.pdf]

# Additional File 1

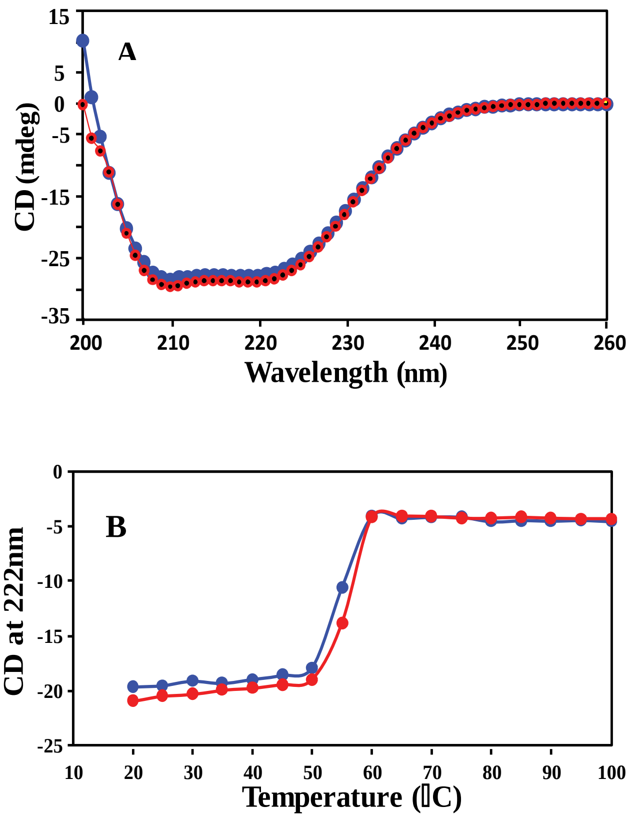


Fig. S1 The CD spectra (A) and thermal melting spectra (B) of VraR (blue solid circles) and VraRM13A (red solid circles).
