# Supplementary material for: The dimerization interface in VraR is essential for induction of the cell wall stress response in Staphylococcus aureus: a potential druggable target
Source: BMC Microbiol. 2019 Jul 5;19:153. doi: 10.1186/s12866-019-1529-0 (PMC6612188; doi:10.1186/s12866-019-1529-0)
Supplement: Supplementary file 2 — DNase I footprinting. Binding isotherms extracted from the DNase I footprinting experiments. (PDF 45 kb) [file 12866_2019_1529_MOESM2_ESM.pdf]

Additional File 2

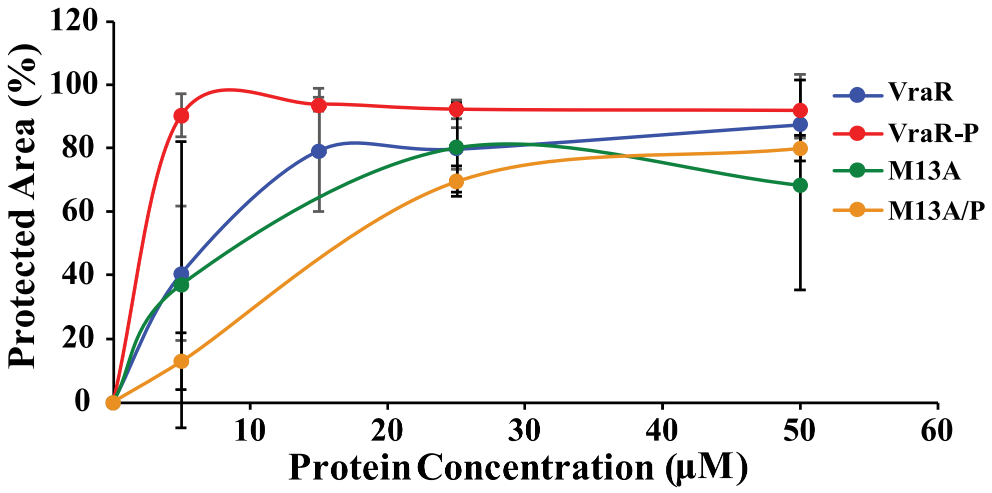

Fig. S2 Binding isotherms of VraR, VraR-P, VraRM13A and acetyl-phosphate-treated VraRM13A (VraRM13A/P). Three DNA bands from the DNase I footprinting gel, located in the VraR-protected region were used to assess VraR binding to DNA. ImageJ was used to quantify the DNA bands.
